# Self-secure feedback control based scheme for ultra-reliable and low-latency communication

**DOI:** 10.1371/journal.pone.0339035

**Published:** 2026-01-09

**Authors:** Zheng Yang, Xiaofang Wang, Shuguang Lu, Jia Wang, Haoheng Yuan

**Affiliations:** 1 Geely University of China, Chengdu, China; 2 School of Intelligent Manufacturing, HongHe Vocational and Technical College, Mengzi, Yunnan, China; 3 School of Information Engineering and Automation, Kunming University of Science and Technology, Kunming, China; Beijing Institute of Technology, CHINA

## Abstract

Ultra-reliable and low-latency communication (URLLC) is one of the key requirements in future wireless communications. In practical URLLC scenarios, an enhanced mobile broadband (eMBB) message together with an URLLC message are simultaneously encoded as codewords and transmitted over the same channels. Traditionally, the coding design of URLLC message often treats the eMBB codeword as interference corrupting the coding performance. In this paper, for the additive white Gaussian noise (AWGN) channel, we show that if noise-free channel feedback is available, there exists a feedback control based coding scheme for the URLLC message, which not only perfectly eliminates the interference caused by the eMBB codeword, but also approaches the maximum rate of the URLLC message when the codeword length tends to infinity. Furthermore, we show that this scheme satisfies the physical layer security requirement by itself, which indicates that our proposed scheme is a self-secure scheme. The results of this paper are explicitly explained by numerical examples, and this work provides a possible way to design efficient coding schemes for URLLC message transmission.

## 1 Introduction

Ultra-reliable and low-latency communications (URLLC) and enhanced mobile broadband (eMBB) are two critical services in 5G and future 6G wireless communications [1–3]. URLLC service (e.g., road safety information and autonomous driving) aims to guarantee high reliability levels, and requires coding scheme with short coding blocklength due to its strict latency constraint. Whereas, eMBB service (e.g., high-resolution video streaming and entertainment applications) aims to provide high transmission rate, and adopts coding scheme with long coding blocklength due to its non-critical latency requirement.

Recently, the coexistence of both URLLC and eMBB services receives much attention. In particular, for the Vehicle-to-Everything (V2X) systems, [4] provided a coexistence mechanism for URLLC and eMBB services, where the arrival URLLC transmission is allowed to puncture eMBB transmission. In addition, [5] studied resource allocation strategy between eMBB and URLLC messages in one-way highway vehicular network. However, note that in practical wireless communication systems, during the eMBB transmission, URLLC messages arrives randomly, and in the meanwhile, the eMBB time slot is divided into mini ones, where the newly arrived URLLC messages are immediately scheduled in the next mini-slot by puncturing the on-going eMBB transmission. In [6], it was shown that treating the eMBB codewords as interference which is non-causally known by the transmitter and applying dirty paper coding scheme, the impact of eMBB codewords on the performance of URLLC messages can be perfectly eliminated. However, note that the proposed scheme in [6] is based on Shannon’s random coding argument, which indicates that the encoding-decoding complexity is too high and the coding blocklength should tend to infinity, and this is unrealistic in practical scenarios. Then it is natural to ask: for the transmission of URLLC message, can we design a low-complexity coding scheme which can also perfectly eliminate the impact of eMBB codeword on the coding performance of the URLLC message? If there does exist such a scheme, can it be a secure scheme satisfying the physical layer security (PLS) requirement, namely, when an external eavesdropper attempts to eavesdropping the URLLC message over an additional noisy channel, no information is leaked to him.

A possible effective solution to the above questions is feedback control based coding scheme, which was first proposed in [7], known as the Schalkwijk-Kailath (SK) scheme. In [7], the additive white Gaussian noise (AWGN) channel with feedback was studied, where the feedback channel is noise free and it helps the transmitter to construct a highly efficient coding scheme. In this scheme, at the first time instant, the message is directly transmitted over the AWGN channel, and the receiver adopts a zero-forcing method to do his first estimation about the message. By noise free channel feedback, the transmitter knows the message’s first estimation by the receiver, and sends the estimation error (difference between the estimation and the real message) at the second time instant. Once receiving the signal, the receiver applies linear minimum mean square estimation (LMMSE) to the received signal and obtains a new estimation about the estimation error at the last time instant, and then he updates his estimation about the message by using this new estimation and the initial estimation. By iteration, it was shown that the receiver’s estimation error about the message vanishes with the grow of the coding blocklength. Later, [8] showed that the SK scheme is in fact a feedback control based scheme, and re-presented this scheme from control-theoretic aspect.

Another interesting property of the SK scheme is that it satisfies the PLS requirement by itself. Here recall that the PLS was first investigated by Shannon [9], and subsequently, Wyner [10] studied how to transmit a message over a noisy channel with perfect secrecy guaranteed. The secrecy capacity, which is the maximum transmission rate with perfect secrecy, was characterized. [11] showed that for the AWGN channel with noise-free feedback and an external eavesdropper, the SK scheme is the optimal secure scheme for such a model, which indicates that the SK scheme not only achieves the optimality, but also is self-secure. This self-secure property of the SK scheme has been extensively studied in literature, see [12,13].

In this paper, we aim to extend the SK scheme to the URLLC message transmission with co-existence of eMBB codewords, and check whether the proposed scheme is self-secure or not. Specifically, for the AWGN channel with noise-free feedback, we propose an SK-type scheme which perfectly eliminates the impact of the eMBB codewords on the coding performance of the URLLC message, and show that the proposed scheme is self-secure. Numerical examples show that our proposed scheme performs well comparing with some existing methods.

*Organization of this paper*: Model formulations and main results are given in Sect 2. Detailed proof of theorems are given in Sect 3. Sect 4 concludes this paper and discusses our possible future research.

For convenience, Table 1 summarizes the notations used in this paper.

**Table 1:** Notations in this paper.

Notation	Meaning
*L*	Codeword length of the entire transmission
*B*	The entire transmission is divided into *B* sub-blocks
*N*	Codeword length of each sub-block
*U*	Total number of occurrences of URLLC message
ρ	Probability of the URLLC message occurrence
ϵ	The given decoding error probability in a sub-block
*P*_*e*,*u*_	The decoding error probability of the *u*-th sub-block (u=1,2,...,U)
*W*_*u*_	Transmitted message in the *u*-th sub-block
$\theta_{u}$	Mapping value of the transmitted message *W*_*u*_
*P*	Power constraints in a sub-block, respectively
$\sigma^{2}$	Noise variance of the feedforward channel
$\sigma_{\tau }^{2}$ and ${\tilde{\sigma }}_{\tau }^{2}$	Noise variances of the eavesdropping channels
$\varepsilon_{u,i}$ and $\alpha_{u,i}$	Estimation error and its variance in the *u*-th sub-block at time instant *i* (i=1,2,...,N)
*R*_*u*_ and $\Delta_{u}$	The achievable rate and secrecy level defined in the *u*-th sub-block
*R* and Δ	The average achievable rate and secrecy level of all URLLC messages
|·|	Cardinality of a set
E(·) and Var(·)	Statistical expectation and variance, respectively
*Q* ^−1^	Converse function of the Gaussion *Q*-function
log	The log function has a base of 2
$𝒩(0,\sigma^{2})$	Gaussian distribution with mean 0 and covariance $\sigma^{2}$

## 2 Model formulation and main results

Assuming a scenario where URLLC and eMBB services coexist under a superposition multiplexing scheme, the receiver wishes to correctly decode eMBB and URLLC messages from the transmitter. The eMBB message arrives at the beginning of a transmission block and is continuously transmitted throughout the whole block, with the block length *L*. On the other hand, to meet the low-latency requirement of URLLC services, the transmission time of URLLC message should be significantly shorter than that of eMBB message, thereby reducing channel occupation duration. Therefore, the eMBB transmission block length is divided into *B* subblocks, each with a length *N*, namely,

L=B·N. (1)

At the start of these subblocks, URLLC message arrives randomly with a probability in ρ∈[0,1]. The transmitter simultaneously sends the eMBB message and newly arrived URLLC message within the subblock. Since eMBB service has non-critical latency requirement, its coding scheme with longer codeword length can be used. In this paper, we assume that the codeword length of eMBB message is sufficiently long and obviously the transmitter knows the eMBB codeword in advance since it is designed by himself, and this indicates that the eMBB codeword can be treated as non-causally known state interference at the transmitter. In the meanwhile, assume that the transmission of URLLC messages occurring *K* times during the entire transmission of eMBB codeword. When eMBB and URLLC messages are superimposed on the same resource for transmission, the receiver identifies whether the current signal contains a URLLC message, and if it does, the receiver feeds back the signal to the transmitter via a feedback channel.

### 2.1 Coexistence of URLLC and eMBB messages transmission over an AWGN channel with feedback

**Fig 1:**
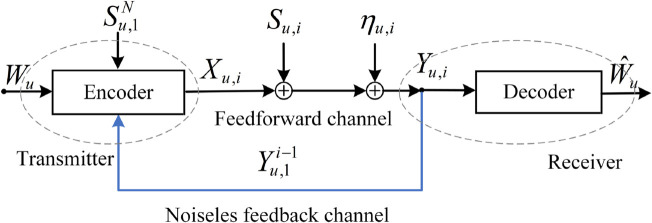
The information-theoretic schematic diagram of the URLLC message transmission in the presence of noiseless feedback. Here *X*_*u*,*i*_ denotes the URLLC codeword at time instant *i* and in the *u*-th (u=1,2,...,U) sub-block, and $S_{u,1}^{N}=(S_{u,1},S_{u,2},...,S_{u,N})$ represents the eMBB codewords in the *u*-th sub-block with blocklength *N*.

The information-theoretic model for the coexistence of URLLC and eMBB transmission with noiseless feedback is shown in Fig 1. In the *u*-th (u=1,2,...,U) sub-block, at time instant *i*, the input-output relationship is given by

$$Y_{u,i}=X_{u,i}+S_{u,i}+\eta_{u,i},\;i=1,2,...,N,$$ (2)

where *X*_*u*,*i*_ is the input of the feedforward channel, *S*_*u*,*i*_ is the codeword of the eMBB message, $\eta_{u,i}\sim 𝒩(0,\sigma^{2})$ is the noise of the AWGN feedforward channel.

**Definition 1:** An $\left(N,|{𝒲}_{u}|,P\right)$-code with power constraint consists of:

(a) The URLLC message *W*_*u*_ in the *u*-th (u=1,2,...,U) sub-block is uniformly distributed over the set ${𝒲}_{u}=\{1,2,...,|{𝒲}_{u}|\}$.

(b) The output of the feedforward encoder is $X_{u,i}=f_{u,i}(W_{u},S_{u,1}^{N},{\tilde{Y}}_{u,1}^{i-1})$ (i=1,2,...,N) and it satisfies the following power constraint

$$\frac{1}{N}\sum_{i=1}^{N}E[X_{u,i}{]}^{2}\leq P,$$ (3)

where $Y_{u,1}^{i}=(Y_{u,1},Y_{u,2},...,Y_{u,i})$.

(c) The output of the receiver’s decoder is ${\hat{W}}_{u}=\psi (Y_{u,1}^{N})$, where ψ is the receiver’s decoding function.

(d) The receiver’s decoding error probability of the URLLC message *W*_*u*_ in the *u*-th (u=1,2,...,U) sub-block is defined as

$$P_{e,u}=\frac{1}{\left|{𝒲}_{u}\right|}\sum_{W_{u}\in {𝒲}_{u}}Pr\{\psi (Y_{u,1}^{N})\neq W_{u}|W_{u}\;\;\text{sent}\},$$ (4)

and the average decoding error probability *P*_*e*_ of all *W*_*u*_ is denoted by

$$P_{e}=\frac{1}{U}\sum_{u=1}^{U}P_{e,u}.$$ (5)

**Definition 2:** The (N,ϵ)-rate *R*_*u*_ is achievable if for given blocklength *N* and decoding error probability ϵ, there exists a $\left(N,|{𝒲}_{u}|,P\right)$-code such that

$$\frac{H(W_{u})}{N}=R_{u},\;\;\;\;P_{e,u}\leq \epsilon .$$ (6)

In (6), the maximum achievable rate *R*_*u*_ is defined as $R_{u}^{*}(N,\epsilon )$, and the capacity *C* is given by

$$C=\lim_{N\to \infty }\lim_{\epsilon \to 0}R_{u}^{⋆}(N,\epsilon ).$$ (7)

Here *R*_*u*_ represents the achievable rate of URLLC message within a single block. According to (1) and (6), the average achievable rate *R* of all URLLC messages is expressed as

$$R=\frac{\sum_{u=1}^{U}H(W_{u})}{L}=\frac{\sum_{u=1}^{U}R_{u}\cdot N}{B\cdot N}=\frac{\sum_{u=1}^{U}R_{u}}{B}.$$ (8)

### 2.2 The model of Fig 1 with an external eavesdropper

**Fig 2:**
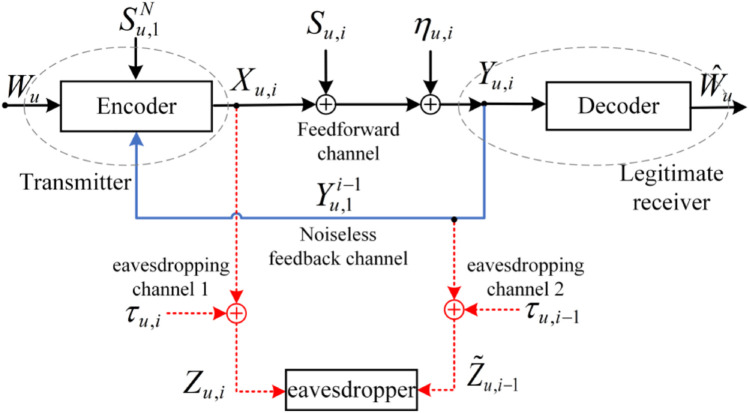
The information-theoretic schematic diagram for the model of Fig 1 with an external eavesdropper.

The information-theoretic schematic diagram for the model of Fig 1 with an external eavesdropper is shown in Fig 2. In the *u*-th (u=1,2,...,U) sub-block, at time instant *i*, the eavesdropper eavesdrops the feedforward and feedback channels by additional AWGN eavesdropping channels, and the input-output relationship of these eavesdropping channels are given by

$$Z_{u,i}=X_{u,i}+\tau_{u,i},\;\;i=1,2,...,N,\;u=1,2,...,U,\;{\tilde{Z}}_{u,i}=Y_{u,i}+{\tilde{\tau }}_{u,i}=X_{u,i}+S_{u,i}+{\tilde{\tau }}_{u,i},$$ (9)

where $\tau_{u,i}\sim 𝒩(0,\sigma_{\tau }^{2})$ and ${\tilde{\tau }}_{u,i}\sim 𝒩(0,{\tilde{\sigma }}_{\tau }^{2})$ are white Gaussian noises and they are independent of each other.

**Definition 3:** Similar to the secrecy criteria defined in [14], in this paper, a normalized eavesdropper’s equivocation (also called secrecy level) for the URLLC message in the *u*-th sub-block is denoted by

$$\Delta_{u}=\frac{H(W_{u}|Z_{u,1}^{N},{\tilde{Z}}_{u,1}^{N-1})}{H(W_{u})},\;0\leq \Delta_{u}\leq 1,$$ (10)

where u=1,2,...,U, $Z_{u,1}^{N}=(Z_{u,1},Z_{u,2},...,Z_{u,N})$ and ${\tilde{Z}}_{u,1}^{N-1}=({\tilde{Z}}_{u,1},{\tilde{Z}}_{u,2},...,{\tilde{Z}}_{u,N-1})$. Here note that $\Delta_{u}$ = 1 corresponds to perfect secrecy, which was first introduced by [9]. The average secrecy level of all URLLC messages

$$\Delta =\frac{\sum_{i=1}^{U}\Delta_{u}}{U}$$ (11)

equals $\Delta_{u}$ since $\Delta_{u}$ of each sub-block remains the same. Hence in this paper, we only need to focus on the security performance in a certain sub-block, namely, $\Delta_{u}$.

### 2.3 Main results

*Theorem 1*: For the model of Fig 1, given decoding error probability ϵ and blocklength *N*, an average achievable rate $R_{avg}(N,\epsilon )$ for all URLLC messages is given by

$$R_{avg}(N,\epsilon )=\rho C-\underset{Losscausedbythegiven(N,\epsilon )}{\underbrace{\frac{\rho }{2N}\log\left(\frac{\left(1+SNR\right){\left[Q^{-1}\left(\frac{\epsilon }{2}\right)\right]}^{2}}{3\cdot SNR}\right)}},$$ (12)

where $SNR=\frac{P}{\sigma^{2}}$ denotes the feedforward signal-to-noise ratio, and *C* is the capacity of the AWGN channel without eMBB codewords [15], which is given by

$$C=\frac{1}{2}\log(1+SNR).$$ (13)

The detailed proof is given in Sect 3.1.

*Remark:* Define the random variable *K*_*u*_ as the effective capacity of the *u*-th sub-block:

$$K_{u}=\left\{ \begin{array}{cc} C_{u}, & \text{with probability }\rho ,\\ 0, & \text{with probability }1-\rho . \end{array}\right.$$ (14)

According to the expected linear properties, the long-term average channel capacity at the system level is given by

$$𝔼[K_{u}]=\rho 𝔼[C_{u}]+(1-\rho )\times 0=\rho 𝔼[C_{u}].$$ (15)

If the signal-to-noise ratio of each transmission sub-block remains constant, according to (13), it can be known that *C*_*u*_ is a fixed constant. Therefore, its expected value $𝔼[C_{u}]$ is equal to itself, that is, $𝔼[C_{u}]=C$. Thus, the system-level long-term average channel capacity of the AWGN channel is ρC. It can be analyzed as the theoretical upper bound on the average achievable rate $R_{avg}(N,\epsilon )$.

The rate expression given by Theorem 1 is similar to that of the finite block-length scenario. Specifically, both of them characterize the actual achievable rate by subtracting the rate loss caused by the given code length and decoding error probability from the channel capacity.

*Theorem 2*: For given decoding error probability ϵ and codeword length *N*, the secrecy level Δ for the model of Fig 2 is lower bounded by

$$\Delta \geq {\left[1-\frac{\log\left(1+\frac{P}{\sigma_{\tau }^{2}}\right)+\log\left(1+\frac{P}{{\tilde{\sigma }}_{\tau }^{2}}\right)}{2NR_{u}(N,\epsilon )}\right]}^{+},$$ (16)

where the function *[x]*^ + ^ = max{x,0},

$$R_{u}(N,\epsilon )=\frac{1}{2}\log\left(1+SNR\right)-\;\frac{1}{2N}\log\left(\frac{\left(1+SNR\right){\left[Q^{-1}\left(\frac{\epsilon }{2}\right)\right]}^{2}}{3\cdot SNR}\right),$$ (17)

$SNR=\frac{P}{\sigma^{2}}$, and $\log(1+\frac{P}{\sigma_{\tau }^{2}})$ and $\log(1+\frac{P}{{\tilde{\sigma }}_{\tau }^{2}})$ in (16) respectively represent the information leakage occurring at the first time instant in feedforward and feedback channels.

The proof is in Sect 3.2.

*Remark:* From (16), we conclude that when the coding blocklength *N* in a certain sub-block *u* tends to infinity, the secrecy level tends to 1, which indicates that our proposed scheme is a self-secure scheme in general.

The following Corollary 1 shows that for a given secrecy threshold δ (0≤δ≤1), the minimum coding blocklength N^*^ such that Δ≥δ is guaranteed.

*Corollary 1*: For given secrecy threshold 0≤δ≤1, decoding error probability ϵ>0, the minimum coding blocklength N^*^ of our proposed scheme satisfying Δ≥δ is given by

$$N^{*}=\frac{A+(1-\delta )\log B}{\left(1-\delta )\log(1+SNR\right)},$$ (18)

where

$$A=\log(1+\frac{P}{\sigma_{\tau }^{2}})+\log(1+\frac{P}{{\tilde{\sigma }}_{\tau }^{2}}),$$ (19)

$$B=\frac{\left(1+SNR\right){\left[Q^{-1}\left(\frac{\epsilon }{2}\right)\right]}^{2}}{3\cdot SNR}.$$ (20)

From Corollary 1, we can easily check that when δ=1 which corresponds to the perfect secrecy, the minimum coding blocklength needs to be infinity.

**Proof:** Letting the lower bound of Theorem 2 satisfy $1-\frac{\log\left(1+\frac{P}{\sigma_{\tau }^{2}}\right)+\log\left(1+\frac{P}{{\tilde{\sigma }}_{\tau }^{2}}\right)}{2NR_{u}(N,\epsilon )}\geq \delta$, the proof of Corollary 1 is completed.

### 2.4 Numerical results

**Fig 3:**
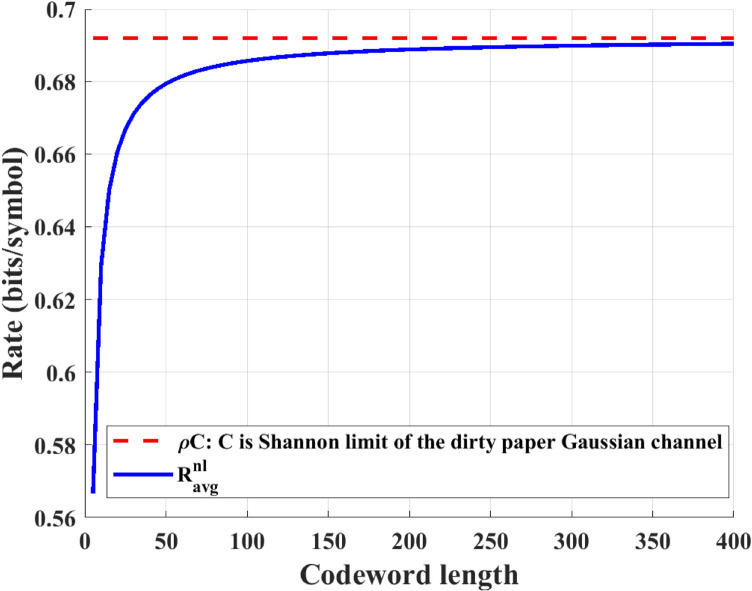
Comparison of the achievable rates $R_{avg}^{nl}(N,\epsilon )$ and ρC under noiseless feedback at different codeword length (ρ = 0.4, P = 10 dB, $\epsilon =10^{-6}$ and $\sigma^{2}$ = 0 dB).

In the experiment setting, we define ρ=0.4, which implies that the URLLC message arrives in a certain subblock with almost equal probability. Besides this, parameters such as *P* = 10*dB* are chosen based on the parameter setting of the experiment section in [5]. Fig 3 compares the achievable rate $R_{avg}^{nl}(N,\epsilon )$ of the proposed scheme and ρC in the case of noiseless feedback. We see that the achievable rate of the proposed scheme almost approaches the Shannon capacity when the codeword length increases.

**Fig 4:**
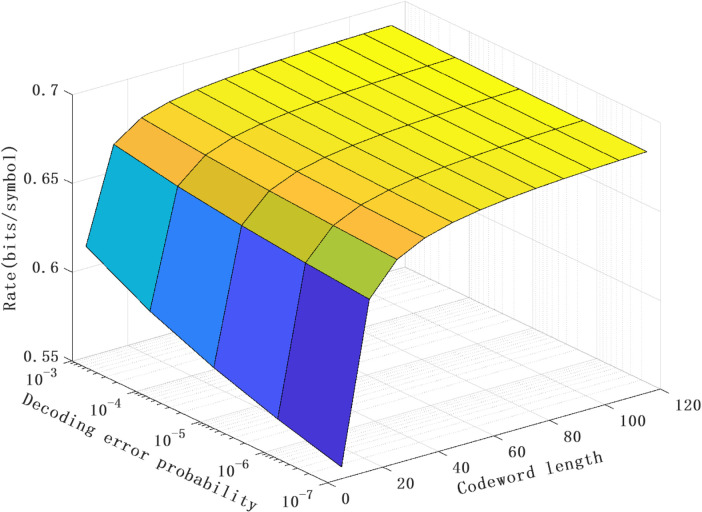
The relationship between the achievable rate $R_{avg}^{nl}(N,\epsilon )$, decoding error probability ϵ and codeword length N of the proposed scheme for the model with noiseless feedback case (ρ = 0.4, P = 10 dB and $\sigma^{2}$ = 0 dB).

Fig 4 plots the relationship between the achievable rate $R_{avg}^{nl}(N,\epsilon )$, the decoding error probability ϵ and the codeword length *N* of the proposed scheme. It can be seen that for fixed codeword length, the achievable rate decreases as the decoding error probability decreases. This indicates that in the process of information transmission, the higher requirement for decoding accuracy, the greater impact on the efficiency of information transmission, consisting with the contradictory relationship between reliability and efficiency. In addition, for fixed decoding error probability, as the codeword length increases, the variation trend of the achievable rate shows that it first rises and then tends to stabilize. This result is in line with the theoretical analysis. According to Theorem 1, under finite-length encoding, the rate loss value caused by (N,ϵ) is negatively correlated with the codeword length. Therefore, in the early stage when the codeword length keeps increasing, the rate loss value continuously decreases, resulting in a corresponding increase in the achievable rate. However, when the codeword length further increases, the rate loss value gradually approaches zero, and the reachable rate also tends to stabilize accordingly. Furthermore, through further observation of Fig 4, it can be known that a lower decoding error probability can be achieved with a shorter codeword length.

**Fig 5:**
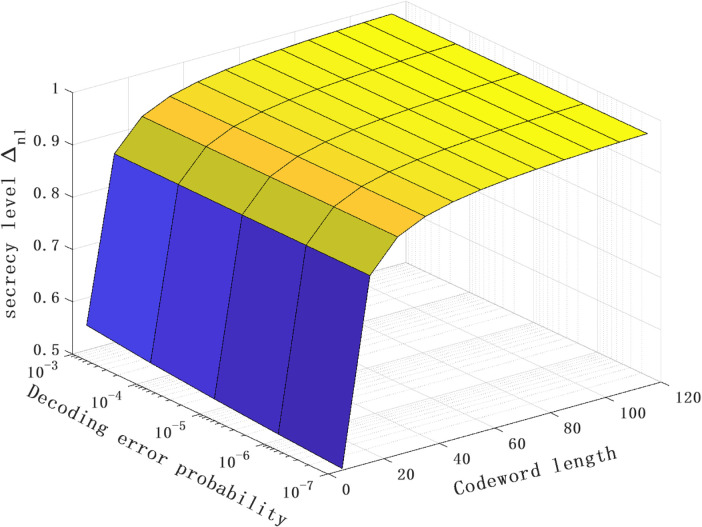
The relationship between the secrecy level $\Delta_{nl}$, decoding error probability ϵ and codeword length N of the proposed scheme for the model with noiseless feedback case (ρ = 0.4, P = 10 dB and $\sigma^{2}=\sigma_{\tau }^{2}={\tilde{\sigma }}_{\tau }^{2}$ = 0 dB).

Fig 5 plots the relationship between the secrecy level $\Delta_{nl}$, decoding error probability ϵ and codeword length *N* of the proposed scheme. It can be seen that when the decoding error probability remains unchanged, the confidentiality level increases with the increase of the codeword length. Specifically, when the decoding error probability is 10^−7^, the codeword length is 50, and the secrecy level is close to 1 (approximately 0.955), this is because in the proposed extended SK coding scheme in a noiseless feedback environment, the feedforward channel and the feedback channel only carry the original transmission message at the first transmission moment, i.e., the information leakage phenomenon only occurs at the first moment. With the continuous increase of the codeword length, the average information leakage of the system gradually decreases. In the meanwhile, as the codeword length tends to infinity, the average information leakage vanishes, indicating that the secrecy level tends to 1, approaching perfect secrecy.

**Fig 6:**
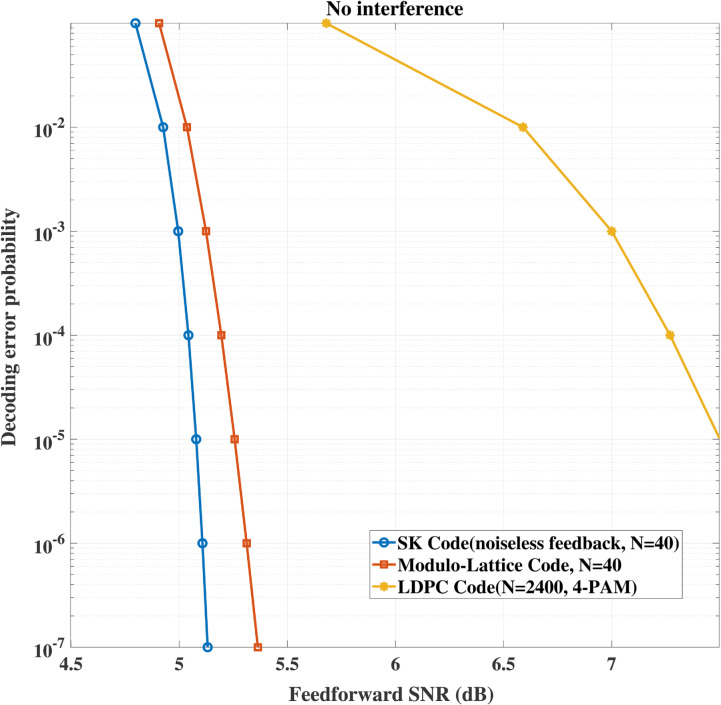
Performance comparison between the LDPC based scheme and the modulo lattice based scheme for ρ = 0.4, $\sigma^{2}$ = 0 dB when no interference involved.

**Fig 7:**
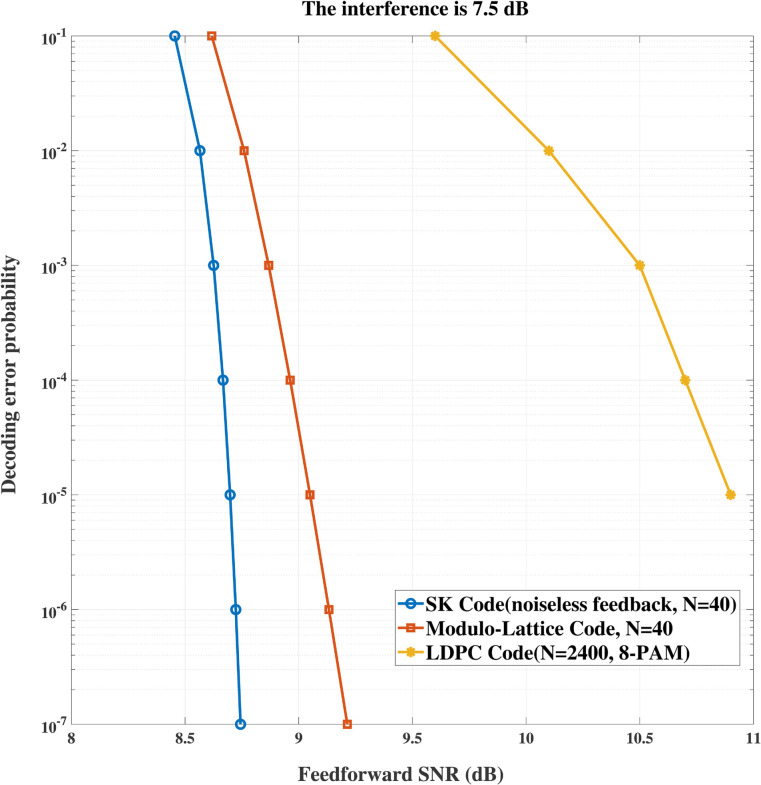
Performance comparison between the LDPC based scheme and the modulo lattice based scheme for ρ = 0.4, $\sigma^{2}$ = 0 dB when interference equals 7.5 dB.

In addition, we compare our proposed SK-type channel coding scheme with LDPC code based dirty paper coding scheme [16] and a modulo lattice based scheme [17]. Figs 6 and 7 show the comparison between the scheme in [16], the modulo lattice based scheme [17] and our proposed scheme for the model with or without interference, respectively. It can be seen that the our scheme gains advantages over the existing schemes. Specifically, when no interference is involved, at the same transmission rate *R* = 1 bits/symbol, to achieve the same decoding error probability, both the codeword length and the feedforward power required by our scheme are smaller than those of existing schemes. This is because the decoding error probability of our scheme decays *doubly exponentially* with the codeword length, while that of existing schemes decays *exponentially* with the increasing of codeword length. On the other hand, when interference is involved and it equals 7.5 dB, it can be seen that our scheme gains advantages over the existing schemes due to the same reason stated above.

## 3 Proof of theorems

### 3.1 Proof of Theorem 1

The intuition behind the proposed scheme of Theorem 1 is that since the transmitter knows all state interference in advance, an estimation offset caused by these state interference in the SK scheme can be computed by the transmitter. Then at the first time instant, if the transmitter inserts a negative value of this estimation offset into the transmission codeword, the entire offset can be perfectly eliminated when the transmission is completed.

**Message mapping:**

For given blocklength *N* and decoding error probabilty ϵ, assume that the URLLC message *W*_*u*_ takes values in the set ${𝒲}_{u}=\{1,2,...,2^{NR_{u}}\}$. The transmitter equally divides the interval $\left[-\frac{1}{2},\frac{1}{2}\right]$ into $2^{NR_{u}}$ sub-intervals, and maps the transmitted message *W*_*u*_ to the midpoint $\theta_{u}$ of sub-interval by defining

$$\theta_{u}=-\frac{1}{2}+\frac{2W_{u}-1}{2\cdot 2^{NR_{u}}}.$$ (21)

Since *W*_*u*_ is uniformly distributed in ${𝒲}_{u}$, $\theta_{u}$ is approximately uniformly distributed over the interval $\left[-\frac{1}{2},\frac{1}{2}\right]$ and its variance is approximately equal to $\frac{1}{12}$, i.e., $E[\theta_{u}{]}^{2}=\frac{1}{12}$.

**Coding procedure:**

At time instant 1, the transmitter encodes

$$X_{u,1}=\sqrt{12P}(\theta_{u}-O),$$ (22)

where

$$O=\frac{S_{u,1}}{\sqrt{12P}}-\sum_{q=2}^{N}\beta_{q}S_{u,q},\;q=2,3,...,N,$$ (23)

and $\beta_{q}$ is the MMSE estimation coefficient (see (30)). Here note that the transmission power at instant 1 is bounded, which is directly from [18].

Once receiving the signal $Y_{u,1}=X_{u,1}+S_{u,1}+\eta_{u,1}=\sqrt{12P}\theta_{u}+\sqrt{12P}\sum_{q=2}^{N}\beta_{q}S_{u,q}+\eta_{u,1}$, the receiver obtains the first estimation ${\hat{\theta }}_{u,1}$ of $\theta_{u}$, i.e.,

$${\hat{\theta }}_{u,1}=\frac{Y_{u,1}}{\sqrt{12P}}=\theta_{u}+\sum_{q=2}^{N}\beta_{q}S_{u,q}+\varepsilon_{u,1},$$ (24)

where the receiver’s estimation error $\varepsilon_{u,1}=\frac{\eta_{1}}{\sqrt{12P}}$, and the variance of $\varepsilon_{u,1}$ is $\alpha_{u,1}=Var(\varepsilon_{u,1})=\frac{\sigma^{2}}{12P}$.

**Iteration:**

At time instant *i*, the transmitter sends

$$X_{u,i}=\sqrt{\frac{P}{\alpha_{u,i-1}}}\varepsilon_{u,i-1},$$ (25)

where the variance of the estimation error $\varepsilon_{u,i-1}$ is $\alpha_{u,i-1}=Var(\varepsilon_{u,i-1})$.

Then the receiver receives $Y_{u,i+1}=X_{u,i+1}+S_{u,i+1}+\eta_{u,i+1}$, and computes the (i+1)-th estimation

$${\hat{\theta }}_{u,i}={\hat{\theta }}_{u,i-1}-\beta_{i}Y_{u,i}={\hat{\theta }}_{u,1}-\sum_{m=2}^{i}\beta_{m}Y_{u,m}\;\overset{\left(a\right)}{=}\theta_{u}+\sum_{q=2}^{N}\beta_{q}S_{u,q}+\varepsilon_{u,1}-\;\;\;\sum_{m=2}^{i}\beta_{m}(X_{u,m}+S_{u,m}+\eta_{u,m})\;\overset{\left(b\right)}{=}\theta_{u}+A_{i+1}+\varepsilon_{u,i}$$ (26)

where (a) follows from $Y_{u,m}=X_{u,m}+S_{u,m}+\eta_{u,m}$ (m=2,3,...,i+1), (b) follows from

$$A_{i+2}=\sum_{q=i+2}^{N}\beta_{q}S_{u,q},$$ (27)

and $\varepsilon_{u,i+1}$ denotes the receiver’s estimation error, which is directly given by

$$\varepsilon_{u,i+1}=\varepsilon_{u,i}-\beta_{i+1}(Y_{u,i+1}-S_{u,i+1})\;=\varepsilon_{u,i}-\beta_{i+1}(X_{u,i+1}+\eta_{u,i+1})\;=\varepsilon_{u,1}-\sum_{m=2}^{i+1}\beta_{m}(X_{u,m}+\eta_{u,m})$$ (28)

the variance of $\varepsilon_{u,i+1}$ is

$$\alpha_{u,i}\overset{\Delta }{**=}\mathrm{Var}(\varepsilon_{u,i})=E[(\varepsilon_{u,i-1}-\beta_{i}(X_{u,i}+\eta_{u,i}){)}^{2}]\;\overset{\left(a\right)}{=}\alpha_{u,i-1}-\frac{P\alpha_{u,i-1}}{P+\sigma^{2}}=\alpha_{u,i-1}\frac{\sigma^{2}}{P+\sigma^{2}}\;=\alpha_{u,1}{\left(\frac{\sigma^{2}}{P+\sigma^{2}}\right)}^{i-1}\overset{\left(b\right)}{=}\frac{\sigma^{2}}{12P}{\left(\frac{\sigma^{2}}{P+\sigma^{2}}\right)}^{i-1},$$ (29)

where (a) follows from $\alpha_{u,i-1}≜Var(\varepsilon_{u,i-1})=E[\varepsilon_{u,i-1}^{2}]$, and the MMSE estimation coefficient

$$\beta_{i}=\frac{E[(Y_{u,i}-S_{u,i})\varepsilon_{u,i-1}]}{E[(Y_{u,i}-S_{u,i}{)}^{2}]}=\frac{E[\varepsilon_{u,i-1}(X_{u,i}+\eta_{u,i})]}{E[(X_{u,i}+\eta_{u,i}{)}^{2}]}\;=\frac{\sqrt{\sigma^{2}}}{\sqrt{12}(P+\sigma^{2})}{\left(\sqrt{\frac{\sigma^{2}}{P+\sigma^{2}}}\right)}^{i-2},\;i=2,...,N.$$ (30)

(b) follows from $\alpha_{u,1}=Var(\varepsilon_{u,1})=\frac{\sigma^{2}}{12P}$.

**End of iteration:**

Finally, From (26), we obtain that the estimation of $\theta_{u}$ at time instant *N* is given by

$${\hat{\theta }}_{u,N}=\theta_{u}+\varepsilon_{u,N},$$ (31)

which is due to the fact that *A*_*N* + 1_ = 0. From (26), we conclude that the receiver’s estimation does not contain any eMBB’s codeword at the final time instant, i.e., the proposed SK-type noiseless feedback scheme eliminates the influence of eMBB message.

**Decoding error probability analysis:**

According to the decoding scheme, the situation where the receiver has a decoding error at the final moment is defined as:

$$E_{N}=\left\{\varepsilon_{u,N}\notin [-\frac{1}{2\cdot 2^{NR_{u}}},\frac{1}{2\cdot 2^{NR_{u}}})\right\}.$$ (32)

From (32), we have

$$Pr(E_{N})=Pr\left\{\varepsilon_{u,N}\notin [-\frac{1}{2\cdot 2^{NR_{u}}},\frac{1}{2\cdot 2^{NR_{u}}})\right\}\;=2Q\left(\frac{1}{2\cdot 2^{NR_{u}}}\cdot \frac{1}{\sqrt{\alpha_{u,N}}}\right)=\epsilon .$$ (33)

Here, Q(·) is the tail function of the Gaussian distribution.

According to (33) and (29), the achievable rate for the noiseless feedback case is given by

$$R_{u}(N,\epsilon )=\frac{1}{2}\log\left(1+SNR\right)-\;\frac{1}{2N}\log\left(\frac{\left(1+SNR\right){\left[Q^{-1}\left(\frac{\epsilon }{2}\right)\right]}^{2}}{3SNR}\right),$$ (34)

where $SNR=\frac{P}{\sigma^{2}}$.

Finally, based on Eqs (8) and (34), the achievable average rate $R_{avg}^{nl}$ in the case of noiseless feedback is:

$$R_{avg}^{nl}(N,\epsilon )=\frac{\sum_{u=1}^{U}R_{u}(N,\epsilon )}{B}\overset{\left(a\right)}{=}\rho \cdot R_{u}(N,\epsilon )\;=\frac{\rho }{2}\log\left(1+SNR\right)-\frac{\rho }{2N}\log\left(\frac{\left(1+SNR\right){\left[Q^{-1}\left(\frac{\epsilon }{2}\right)\right]}^{2}}{3SNR}\right)$$ (35)

where (a) follows from $\rho =\frac{U}{B}$, which is realized when the number of sub-blocks is sufficiently large, i.e., the law of large numbers. Thus completing the proof of Theorem 1.

### 3.2 Proof of Theorem 2

The intuition behind the security analysis is that the transmitted message of our proposed scheme is only involved into the codeword sequence at the very beginning, which indicates that information leakage only occurs at the first time instant, resulting in the average information leakage vanishes as the coding blocklength tends to infinity.

To analyze the secrecy level defined in (10), $H(W_{u}|Z_{u,1}^{N},{\tilde{Z}}_{u,1}^{N-1})$ is first bounded by

$$\;H(W_{u}|Z_{u,1}^{N},{\tilde{Z}}_{u,1}^{N-1})\;\overset{\left(a\right)}{**\geq }H(\theta_{u}|Z_{u,1}^{N},{\tilde{Z}}_{u,1}^{N-1},\eta_{u,1}^{N},\tau_{u,2}^{N},{\tilde{\tau }}_{u,2}^{N-1},S_{u,1}^{N})\;\overset{\left(b\right)}{=}H(\theta_{u}|\underset{Z_{u,1}}{\underbrace{X_{u,1}+\tau }},\ldots ,\underset{Z_{u,N}}{\underbrace{X_{u,N}+\tau_{u,N}}},\;\;\underset{{\tilde{Z}}_{u,1}}{\underbrace{Y_{u,1}+\tilde{\tau }}},\ldots ,\underset{{\tilde{Z}}_{u,N-1}}{\underbrace{Y_{u,N-1}+{\tilde{\tau }}_{u,N-1}}},\eta_{u,1}^{N},\tau_{u,2}^{N},{\tilde{\tau }}_{u,2}^{N-1},S_{u,1}^{N})\;\overset{\left(c\right)}{**=}H(\theta_{u}|\sqrt{12P}(\theta_{u}-O)+\tau_{u,1},\sqrt{12P}(\theta_{u}-O)+{\tilde{\tau }}_{u,1},\;\tau_{u,2}^{N},{\tilde{\tau }}_{u,2}^{N-1},S_{u,1}^{N})\;\overset{\left(d\right)}{**=}H(\theta_{u}|\sqrt{12P}\theta_{u}+\tau_{u,1},\sqrt{12P}\theta_{u}+{\tilde{\tau }}_{u,1})\;\overset{\left(e\right)}{**\geq }H(\theta_{u})+h(\tau_{u,1})+h({\tilde{\tau }}_{u,1})-\;[h(\sqrt{12P}\theta_{u}+\tau_{u,1})+h(\sqrt{12P}\theta_{u}+{\tilde{\tau }}_{u,1})]\;\overset{\left(f\right)}{**\geq }H(\theta_{u})\underset{\text{The information leakage that occurred at time 1}}{\underbrace{-\frac{1}{2}log(1+\frac{P}{\sigma_{\tau }^{2}})-\frac{1}{2}log(1+\frac{P}{{\tilde{\sigma }}_{\tau }^{2}})}}$$ (36)

where

(a) follows from conditioning reduces entropy,

(b) follows from (9),

(c) follows from that the code word $X_{u,1}=\sqrt{12P}(\theta_{u}\;-\;O)$ sent at the first moment and the code word $X_{u,i}(i=2,3,\cdots ,N)$ sent at the $X_{u,1}=\sqrt{12P}(\theta_{u}\;-\;O)$ moment are functions of the estimation error (channel noise $\eta_{u,1}^{N}$), and $\left(S_{u,1}^{N},\eta_{u,1}^{N}\right)$ and $\left(\theta_{u},\tau_{u,1}^{N},{\tilde{\tau }}_{u,1}^{N-1}\right)$ in $Y_{u,i}=X_{u,i}+S_{u,i}+\eta_{u,1}^{N}$ are independent of each other,

(d) follows from (22) that $X_{u,1}=\sqrt{12P}(\theta_{u}-O)$, here *O* is a function of $S_{u,1}^{N}$,

(e) follows from the fact that h(X|Y,Z)=h(X,Y,Z)−h(Y,Z)≥h(X,Y,Z)−(h(Y)+h(Z)),

(f) follows from that the fact that

$$h(\tau_{u,1})=\frac{1}{2}\log(2\pi e\sigma_{\tau }^{2})\;h({\tilde{\tau }}_{u,1})=\frac{1}{2}\log(2\pi e{\tilde{\sigma }}_{\tau }^{2})\;h(\sqrt{12P}\theta +\tau_{\mu ,1})\leq \frac{1}{2}\log(2\pi e(P+\sigma_{\tau }^{2}))\;h(\sqrt{12P}\theta +{\tilde{\tau }}_{u,1})\leq \frac{1}{2}\log(2\pi e(P+{\tilde{\sigma }}_{\tau }^{2})).$$ (37)

From (36) and the fact that $H(W_{u})=H(\theta_{u})=NR_{u}(N,\epsilon )$, we conclude that

$$\Delta_{u}=\frac{H(W_{u}|Z_{u,1}^{N},{\tilde{Z}}_{u,1}^{N-1})}{H(W_{u})}\;\geq 1-\frac{\log(1+\frac{P}{\sigma_{\tau }^{2}})+\log(1+\frac{P}{{\tilde{\sigma }}_{\tau }^{2}})}{2NR_{u}(N,\epsilon )}.$$ (38)

Therefore, the average secrecy level of the overall URLLC message can be expressed as

$$\Delta =\Delta_{u}\;\geq 1-\frac{\log(1+\frac{P}{\sigma_{\tau }^{2}})+\log(1+\frac{P}{{\tilde{\sigma }}_{\tau }^{2}})}{2NR_{u}(N,\epsilon )},$$ (39)

where $R_{u}(N,\epsilon )$ is given in (34). Since Δ≥0, the proof of Theorem 2 is completed.

## 4 Conclusion and future work

In this paper, the URLLC message transmission with co-existence of eMBB codewords is investigated. Specifically, for the AWGN channel with noise-free feedback, we propose a feedback control based coding scheme for the URLLC message, which not only perfectly eliminates the interference caused by the eMBB codeword, but also approaches the maximum rate of the URLLC message when the codeword length tends to infinity. Furthermore, we show that this scheme satisfies the physical layer security requirement by itself. One possible future work is to extend the proposed scheme to fading and MIMO channels. Another interesting problem is to check whether the SK-type scheme is suitable for the covert communication or not, and if so, how to achieve positive rate of covert communication by the SK-type scheme, like [19] does.

## Supporting information

S1 Text Detailed proof of theorems. (PDF)
