# Supplementary material for: Self-secure feedback control based scheme for ultra-reliable and low-latency communication
Source: PLoS One. 2026 Jan 9;21(1):e0339035. doi: 10.1371/journal.pone.0339035 (PMC12788644; doi:10.1371/journal.pone.0339035)
Supplement: S1 Text — (PDF) [file pone.0339035.s001.pdf]

1 Proof of theorems

1.1 Proof of Theorem 1

The intuition behind the proposed scheme of Theorem 1 is that since the transmitter knows all state interference in advance, an estimation offset caused by these state interference in the SK scheme can be computed by the transmitter. Then at the first time instant, if the transmitter inserts a negative value of this estimation offset into the transmission codeword, the entire offset can be perfectly eliminated when the transmission is completed.

Message mapping:

For given blocklength N and decoding error probabilty ϵ, assume that the URLLC message W_u takes values in the set $\mathcal{W}_u = \{1, 2, ..., 2^{NR_u}\}$. The transmitter equally divides the interval $[-\frac{1}{2}, \frac{1}{2}]$ into 2^{NR_u} sub-intervals, and maps the transmitted message W_u to the midpoint θ_u of sub-interval by defining

$$\theta_u = -\frac{1}{2} + \frac{2W_u - 1}{2 \cdot 2^{NR_u}}. \tag{1}$$

Since W_u is uniformly distributed in $\mathcal{W}_u$, θ_u is approximately uniformly distributed over the interval $[-\frac{1}{2}, \frac{1}{2}]$ and its variance is approximately equal to $\frac{1}{12}$, i.e., $E[\theta_u]^2 = \frac{1}{12}$.

Coding procedure:

At time instant 1, the transmitter encodes

$$X_{u,1} = \sqrt{12P}(\theta_u - O), \tag{2}$$

where

$$O = \frac{S_{u,1}}{\sqrt{12P}} - \sum_{q=2}^{N} \beta_q S_{u,q}, \quad q = 2, 3, ..., N, \tag{3}$$

and β_q is the MMSE estimation coefficient (see (10)). Here note that the transmission power at instant 1 is bounded, which is directly from [18].

Once receiving the signal $Y_{u,1} = X_{u,1} + S_{u,1} + \eta_{u,1} = \sqrt{12P}\theta_u + \sqrt{12P}\sum_{q=2}^{N} \beta_q S_{u,q} + \eta_{u,1}$, the receiver obtains the first estimation $\widehat{\theta}_{u,1}$ of θ_u, i.e.,

$$\widehat{\theta}_{u,1} = \frac{Y_{u,1}}{\sqrt{12P}} = \theta_u + \sum_{q=2}^{N} \beta_q S_{u,q} + \varepsilon_{u,1}, \tag{4}$$

where the receiver's estimation error $\varepsilon_{u,1} = \frac{\eta_1}{\sqrt{12P}}$, and the variance of $\varepsilon_{u,1}$ is $\alpha_{u,1} = Var(\varepsilon_{u,1}) = \frac{\sigma^2}{12P}$.

Iteration:

At time instant i, the transmitter sends

$$X_{u,i} = \sqrt{\frac{P}{\alpha_{u,i-1}}}\varepsilon_{u,i-1}, \tag{5}$$

where the variance of the estimation error $\varepsilon_{u,i-1}$ is $\alpha_{u,i-1} = \mathrm{Var}(\varepsilon_{u,i-1})$.

Then the receiver receives $Y_{u,i+1} = X_{u,i+1} + S_{u,i+1} + \eta_{u,i+1}$, and computes the $(i+1)$-th estimation

$$\begin{aligned}\hat{\theta}_{u,i} &= \hat{\theta}_{u,i-1} - \beta_i Y_{u,i} = \hat{\theta}_{u,1} - \sum_{m=2}^{i} \beta_m Y_{u,m} \\ &\overset{(a)}{=} \theta_u + \sum_{q=2}^{N} \beta_q S_{u,q} + \varepsilon_{u,1} - \\ &\sum_{m=2}^{i} \beta_m (X_{u,m} + S_{u,m} + \eta_{u,m}) \\ &\overset{(b)}{=} \theta_u + A_{i+1} + \varepsilon_{u,i}\end{aligned} \tag{6}$$

where (a) follows from $Y_{u,m} = X_{u,m} + S_{u,m} + \eta_{u,m}$ $(m = 2, 3, ..., i+1)$, (b) follows from

$$A_{i+2} = \sum_{q=i+2}^{N} \beta_q S_{u,q}, \tag{7}$$

and $\varepsilon_{u,i+1}$ denotes the receiver's estimation error, which is directly given by

$$\begin{aligned}\varepsilon_{u,i+1} &= \varepsilon_{u,i} - \beta_{i+1}(Y_{u,i+1} - S_{u,i+1}) \\ &= \varepsilon_{u,i} - \beta_{i+1}(X_{u,i+1} + \eta_{u,i+1}) \\ &= \varepsilon_{u,1} - \sum_{m=2}^{i+1} \beta_m (X_{u,m} + \eta_{u,m})\end{aligned} \tag{8}$$

the variance of $\varepsilon_{u,i+1}$ is

$$\begin{aligned}\alpha_{u,i} &\triangleq \mathrm{Var}(\varepsilon_{u,i}) = E[(\varepsilon_{u,i-1} - \beta_i (X_{u,i} + \eta_{u,i}))^2] \\ &\overset{(a)}{=} \alpha_{u,i-1} - \frac{P\alpha_{u,i-1}}{P+\sigma^2} = \alpha_{u,i-1} \frac{\sigma^2}{P+\sigma^2} \\ &= \alpha_{u,1} \left(\frac{\sigma^2}{P+\sigma^2}\right)^{i-1} \overset{(b)}{=} \frac{\sigma^2}{12P} \left(\frac{\sigma^2}{P+\sigma^2}\right)^{i-1},\end{aligned} \tag{9}$$

where (a) follows from $\alpha_{u,i-1} \triangleq \mathrm{Var}(\varepsilon_{u,i-1}) = E[\varepsilon_{u,i-1}^2]$, and the MMSE estimation coefficient

$$\begin{aligned}\beta_i &= \frac{E[(Y_{u,i} - S_{u,i})\varepsilon_{u,i-1}]}{E[(Y_{u,i} - S_{u,i})^2]} = \frac{E[\varepsilon_{u,i-1}(X_{u,i} + \eta_{u,i})]}{E[(X_{u,i} + \eta_{u,i})^2]} \\ &= \frac{\sqrt{\sigma^2}}{\sqrt{12}(P+\sigma^2)} \left(\sqrt{\frac{\sigma^2}{P+\sigma^2}}\right)^{i-2}, \quad i = 2, ..., N.\end{aligned} \tag{10}$$

(b) follows from $\alpha_{u,1} = \mathrm{Var}(\varepsilon_{u,1}) = \frac{\sigma^2}{12P}$.

End of iteration:

Finally, From (6), we obtain that the estimation of θ_u at time instant N is given by

$$\widehat{\theta}_{u,N} = \theta_u + \varepsilon_{u,N}, \tag{11}$$

which is due to the fact that $A_{N+1} = 0$. From (6), we conclude that the receiver's estimation does not contain any eMBB's codeword at the final time instant, i.e., the proposed SK-type noiseless feedback scheme eliminates the influence of eMBB message.

Decoding error probability analysis:

According to the decoding scheme, the situation where the receiver has a decoding error at the final moment is defined as:

$$E_N = \left\{ \varepsilon_{u,N} \notin [-\frac{1}{2 \cdot 2^{NR_u}}, \frac{1}{2 \cdot 2^{NR_u}}) \right\}. \tag{12}$$

From (12), we have

$$\begin{aligned} Pr(E_N) &= Pr\left\{ \varepsilon_{u,N} \notin [-\frac{1}{2 \cdot 2^{NR_u}}, \frac{1}{2 \cdot 2^{NR_u}}) \right\} \\ &= 2Q\left(\frac{1}{2 \cdot 2^{NR_u}} \cdot \frac{1}{\sqrt{\alpha_{u,N}}} \right) = \epsilon. \end{aligned} \tag{13}$$

Here, $Q(\cdot)$ is the tail function of the Gaussian distribution.

According to (13) and (9), the achievable rate for the noiseless feedback case is given by

$$\begin{aligned} R_u(N,\epsilon) = \frac{1}{2}\log(1+SNR) - \\ \frac{1}{2N}\log\left(\frac{(1+SNR)\left[Q^{-1}\left(\frac{\epsilon}{2}\right)\right]^2}{3SNR} \right), \end{aligned} \tag{14}$$

where $SNR = \frac{P}{\sigma^2}$.

Finally, based on equation (14), the achievable average rate R_{avg}^{nl} in the case of noiseless feedback is:

$$\begin{aligned} R_{avg}^{nl}(N,\epsilon) = \frac{\sum_{u=1}^{U} R_u(N,\epsilon)}{B} \overset{(a)}{=} \rho \cdot R_u(N,\epsilon) \\ = \frac{\rho}{2}\log(1+SNR) - \frac{\rho}{2N}\log\left(\frac{(1+SNR)\left[Q^{-1}\left(\frac{\epsilon}{2}\right)\right]^2}{3SNR} \right) \end{aligned} \tag{15}$$

where (a) follows from $\rho = \frac{U}{B}$, which is realized when the number of sub-blocks is sufficiently large, i.e., the law of large numbers. Thus completing the proof of Theorem 1.

1.2 Proof of Theorem 2

The intuition behind the security analysis is that the transmitted message of our proposed scheme is only involved into the codeword sequence at the very beginning, which indicates that information leakage only occurs at the first time instant, resulting in the average information leakage vanishes as the coding blocklength tends to infinity.

To analyze the secrecy level defined in Definition 3, $H(W_u|Z_{u,1}^N, \widetilde{Z}_{u,1}^{N-1})$ is first

bounded by

$$
\begin{aligned}
& H(W_u \mid Z_{u,1}^N, \tilde{Z}_{u,1}^{N-1}) \\
& \overset{(a)}{\geq} H(\theta_u \mid Z_{u,1}^N, \tilde{Z}_{u,1}^{N-1}, \eta_{u,1}^N, \tau_{u,2}^N, \tilde{\tau}_{u,2}^{N-1}, S_{u,1}^N) \\
& \overset{(b)}{=} H\big(\theta_u \mid \underbrace{X_{u,1} + \tau}_{Z_{u,1}}, \ldots, \underbrace{X_{u,N} + \tau_{u,N}}_{Z_{u,N}}, \\
& \underbrace{Y_{u,1} + \tilde{\tau}}_{\tilde{Z}_{u,1}}, \ldots, \underbrace{Y_{u,N-1} + \tilde{\tau}_{u,N-1}}_{\tilde{Z}_{u,N-1}}, \eta_{u,1}^N, \tau_{u,2}^N, \tilde{\tau}_{u,2}^{N-1}, S_{u,1}^N\big) \\
& \overset{(c)}{=} H(\theta_u \mid \sqrt{12P}(\theta_u - O) + \tau_{u,1}, \sqrt{12P}(\theta_u - O) + \tilde{\tau}_{u,1}, \\
& \tau_{u,2}^N, \tilde{\tau}_{u,2}^{N-1}, S_{u,1}^N) \\
& \overset{(d)}{=} H(\theta_u \mid \sqrt{12P}\theta_u + \tau_{u,1}, \sqrt{12P}\theta_u + \tilde{\tau}_{u,1}) \\
& \overset{(e)}{\geq} H(\theta_u) + h(\tau_{u,1}) + h(\tilde{\tau}_{u,1}) - \\
& [h(\sqrt{12P}\theta_u + \tau_{u,1}) + h(\sqrt{12P}\theta_u + \tilde{\tau}_{u,1})] \\
& \overset{(f)}{\geq} H(\theta_u) \quad \underbrace{-\frac{1}{2}\mathbf{log}(1 + \frac{P}{\sigma_\tau^2}) - \frac{1}{2}\mathbf{log}(1 + \frac{P}{\tilde{\sigma}_\tau^2})}_{\text{The information leakage that occurred at time 1}}
\end{aligned}
\tag{16}
$$

where

(a) follows from conditioning reduces entropy,

(b) follows from that $Z_{u,i} = X_{u,i} + \tau_{u,i}$ and $\widetilde{Z}_{u,i} = Y_{u,i} + \widetilde{\tau}_{u,i} = X_{u,i} + S_{u,i} + \widetilde{\tau}_{u,i}$, where $i = 1, 2, ..., N, \quad u = 1, 2, ..., U$.

(c) follows from that the code word $X_{u,1} = \sqrt{12P}(\theta_u - O)$ sent at the first moment and the code word $X_{u,i}(i = 2, 3, \cdots, N)$ sent at the $X_{u,1} = \sqrt{12P}(\theta_u - O)$ moment are functions of the estimation error (channel noise $\eta_{u,1}^N$), and $(S_{u,1}^N, \eta_{u,1}^N)$ and $(\theta_u, \tau_{u,1}^N, \tilde{\tau}_{u,1}^{N-1})$ in $Y_{u,i} = X_{u,i} + S_{u,i} + \eta_{u,1}^N$ are independent of each other,

(d) follows from (2) that $X_{u,1} = \sqrt{12P}(\theta_u - O)$, here O is a function of $S_{u,1}^N$,

(e) follows from the fact that
$h(X|Y, Z) = h(X, Y, Z) - h(Y, Z) \geq h(X, Y, Z) - (h(Y) + h(Z))$,

(f) follows from that the fact that

$$
\begin{aligned}
h(\tau_{u,1}) &= \frac{1}{2}\log(2\pi e \sigma_\tau^2) \\
h(\tilde{\tau}_{u,1}) &= \frac{1}{2}\log(2\pi e \tilde{\sigma}_\tau^2) \\
h(\sqrt{12P}\theta + \tau_{\mu,1}) &\leq \frac{1}{2}\log(2\pi e(P + \sigma_\tau^2)) \\
h(\sqrt{12P}\theta + \tilde{\tau}_{u,1}) &\leq \frac{1}{2}\log(2\pi e(P + \tilde{\sigma}_\tau^2)).
\end{aligned}
\tag{17}
$$

From (16) and the fact that $H(W_u) = H(\theta_u) = NR_u(N, \epsilon)$, we conclude that

$$
\begin{aligned}
\Delta_u &= \frac{H(W_u|Z_{u,1}^N, \widetilde{Z}_{u,1}^{N-1})}{H(W_u)} \\
&\geq 1 - \frac{\log(1 + \frac{P}{\sigma_\tau^2}) + \log(1 + \frac{P}{\tilde{\sigma}_\tau^2})}{2NR_u(N, \epsilon)}.
\end{aligned}
\tag{18}
$$

Therefore, the average secrecy level of the overall URLLC message can be expressed as

$$\begin{aligned}\Delta &= \Delta_u \\ &\geq 1 - \frac{\log(1+\frac{P}{\sigma_\tau^2}) + \log(1+\frac{P}{\tilde{\sigma}_\tau^2})}{2NR_u(N,\epsilon)},\end{aligned} \tag{19}$$

where $R_u(N,\epsilon)$ is given in (14). Since $\Delta \geq 0$, the proof of Theorem 2 is completed.

References

1. Ge X. Ultra-reliable low-latency communications in autonomous vehicular networks. IEEE Transactions on Vehicular Technology. 2019; 68(5):5005-5016.

2. Tang J, Shim B, Quek TQS. Service multiplexing and revenue maximization in sliced C-RAN incorporated with URLLC and multicast eMBB. IEEE Journal on Selected Areas in Communications. 2019; 37(4):881-895.

3. Anand A, Veciana GD, Shakkottai S. Joint scheduling of URLLC and eMBB traffic in 5G wireless networks. IEEE/ACM Transactions on Networking. 2020; 28(2):477-490.

4. Kassab R, Simeone O, Popovski P. Coexistence of URLLC and eMBB services in the C-RAN uplink: An information-theoretic study. In: 2018 IEEE Global Communications Conference (GLOBECOM); 2018. p. 1-6.

5. Anand A, Veciana GD. Resource allocation and HARQ optimization for URLLC traffic in 5G wireless networks. IEEE Journal on Selected Areas in Communications. 2018; 36(11):2411-2421.

6. Nikbakht H, Ruzomberka E, Wigger M, Shitz SS, Poor HV. Joint coding of eMBB and URLLC in vehicle-to-everything (V2X) communications. In: GLOBECOM 2023 - 2023 IEEE Global Communications Conference; 2023. p. 1-6.

7. Schalkwijk J, Kailath T. A coding scheme for additive noise channels with feedback–I: No bandwidth constraint. IEEE Transactions on Information Theory. 1966; 12(2):172-182.

8. Elia N. When Bode meets Shannon: Control-oriented feedback communication schemes. IEEE Transactions on Automatic Control. 2004; 49(9):1477-1488.

9. Shannon CE. Communication theory of secrecy systems. The Bell System Technical Journal. 1949; 28(4):656-715.

10. Wyner AD. The wire-tap channel. The Bell System Technical Journal. 1975; 54(8):1355-1387.

11. Gündüz D, Brown DR, Poor HV. Secret communication with feedback. In: International Symposium on Information Theory and Its Applications (ISITA); 2008. p. 1-6.

12. Dai B, Li C, Liang Y, Ma Z, Shamai S. Self-secure capacity-achieving feedback schemes of Gaussian multiple-access wiretap channels with degraded message sets. IEEE Transactions on Information Forensics and Security. 2022; 17:1583-1596.

13. Li C, Liang Y, Poor HV, Shamai S. Secrecy capacity of colored Gaussian noise channels with feedback. IEEE Transactions on Information Theory. 2019; 65(9):5771-5782.

14. Tekin E, Yener A. The Gaussian multiple access wire-tap channel. IEEE Transactions on Information Theory. 2008; 54(12):5747-5755.

15. Costa M. Writing on dirty paper (Corresp.). IEEE Transactions on Information Theory. 1983; 29(3):439-441.

16. Rege KM, Balachandran K, Kang JH, Karakayali MK. Practical dirty paper coding with sum codes. IEEE Transactions on Communications. 2016; 64(2):441-455.

17. Ben-Yishai A, Shayevitz O. Interactive schemes for the AWGN channel with noisy feedback. IEEE Transactions on Information Theory. 2017; 63(4):2409-2427.

18. Rosenzweig A. The capacity of Gaussian multi-user channels with state and feedback. IEEE Transactions on Information Theory. 2007; 53(11):4349-4355.

19. Kang B, Ye N, An J. Achieving positive rate of covert communications covered by randomly activated overt users. IEEE Transactions on Information Forensics and Security. 2025; 20:2480-2495.
